# Improvement of Pro-Oxidant Capacity of Protocatechuic Acid by Esterification

**DOI:** 10.1371/journal.pone.0110277

**Published:** 2014-10-23

**Authors:** Maria Luiza Zeraik, Maicon S. Petrônio, Dyovani Coelho, Luis Octavio Regasini, Dulce H. S. Silva, Luiz Marcos da Fonseca, Sergio A. S. Machado, Vanderlan S. Bolzani, Valdecir F. Ximenes

**Affiliations:** 1 Department of Chemistry, Faculty of Sciences, São Paulo State University (UNESP), Bauru, São Paulo, Brazil; 2 Department of Organic Chemistry, Nuclei of Bioassays, Ecophysiology and Biosynthesis of Natural Products (NuBBE), Institute of Chemistry, São Paulo State University (UNESP), Araraquara, São Paulo, Brazil; 3 Department of Clinical Analysis, School of Pharmaceutical Sciences, São Paulo State University (UNESP), Araraquara, São Paulo, Brazil; 4 Institute of Chemistry of São Carlos, São Paulo University (USP), São Carlos, SP, Brazil; UMR INSERM U866, France

## Abstract

Pro-oxidant effects of phenolic compounds are usually correlated to the one-electron redox potential of the phenoxyl radicals. Here we demonstrated that, besides their oxidizability, hydrophobicity can also be a decisive factor. We found that esterification of protocatechuic acid (P0) provoked a profound influence in its pro-oxidant capacity. The esters bearing alkyl chains containing two (P2), four (P4) and seven (P7) carbons, but not the acid precursor (P0), were able to exacerbate the oxidation of trolox, α-tocopherol and rifampicin. This effect was also dependent on the catechol moiety, since neither gallic acid nor butyl gallate showed any pro-oxidant effects. A comparison was also made with apocynin, which is well-characterized regarding its pro-oxidant properties. P7 was more efficient than apocynin regarding co-oxidation of trolox. However, P7 was not able to co-oxidize glutathione and NADH, which are targets of the apocynin radical. A correlation was found between pro-oxidant capacity and the stability of the radicals, as suggested by the intensity of the peak current in the differential pulse voltammetry experiments. In conclusion, taking into account that hydroquinone and related moieties are frequently found in biomolecules and quinone-based chemotherapeutics, our demonstration that esters of protocatechuic acid are specific and potent co-catalysts in their oxidations may be very relevant as a pathway to exacerbate redox cycling reactions, which are usually involved in their biological and pharmacological mechanisms of action.

## Introduction

A pro-oxidant effect is a general term used when biological, chemical or physical events are able to initiate or exacerbate, by different mechanisms, a redox imbalance. It can be directly or indirectly related to the generation of reactive oxygen species (ROS) or to the inhibition of endogenous protective antioxidant systems. It is important to note that a pro-oxidant effect is the opposite of an antioxidant effect, but not necessarily related to deleterious and pathological consequences. Indeed, there are numerous and growing evidences that compounds of natural or synthetic origin usually accepted as classical antioxidants can also act as pro-oxidants. In this regard, one of the most well-established substances is the potent antioxidant ascorbic acid, which besides its antiradical capacity is also able to initiate a pro-oxidant reaction by reducing ferric ion Fe(III) to ferrous ion Fe(II), the first step in the generation of the hydroxyl radical (HO•) through the Fenton reaction [1]. Moreover, it is common that their pro-oxidant properties are involved in their beneficial biological effects. This is the case with ascorbic acid and vitamin K_3_, which are the active components in Apatone, a drug combination that has been used to kill tumour cells. The mechanism for the pharmacological action of Apatone is linked to the redox cycling of ascorbic acid, which induces a pro-oxidant effect and provokes the death of tumour cells by autoschizis [2].

The pro-oxidant activity of polyphenols is usually related to the generation of ROS, in the form of superoxide anions (O_2_^•-^), hydrogen peroxide (H_2_O_2_) and peroxyl radicals (ROO•), being the last the intermediate species in lipid peroxidation [3]–[7]. These oxidants are produced by autoxidation reactions, i.e. their direct oxidation by molecular oxygen [8]–[10]. The typical mechanism of polyphenol autoxidation reactions is demonstrated in the equations below (eq. 1–2). Additionally, these active redox molecules are also able to reduce Fe(III) to Fe(II), hence enabling the production of HO• (eq. 3–4) [11]. Consistent with that, lipoperoxidation, DNA damage, inactivation of antioxidant enzymes, mitochondrial disruption and other ROS-mediated deleterious processes are frequently associated with the antiproliferative effects and induction of apoptosis associated with the use of these molecules [3]–[6].
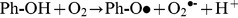
(1)

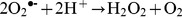
(2)

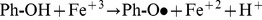
(3)

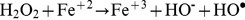
(4)


Pro-oxidant activity has also been demonstrated for phenolic acids such as gallic, caffeic, ferulic acids and their derivatives. For instance, the cytoxicity of caffeic and gallic acids and their esters against human promyelocytic leukaemia is associated with both pro-oxidant activity and lipophilicity [12]; gallic acid increases ROS levels as well as depleting glutathione in A549 lung cancer cells [13]; caffeic acid phenylethyl ester induces apoptosis of human leukaemic cells by disrupting mitochondrial function via a pathway related to an increase in intracellular ROS [14]; caffeic acid, ferulic acid and their derivatives are effective agents for cleaving DNA in the presence of Cu(II) [15].

Another phytochemical that has been widely studied is protocatechuic acid, a natural phenolic compound found in many edible and medicinal plants. Indeed, besides its antiradical activity, protocatechuic acid has analgesic and anti-inflammatory properties that are comparable with those of standard drugs [16]. Recently, we demonstrated that the esterification of protocatechuic acid increased its efficiency as an inhibitor of the multienzymatic complex NADPH oxidase in neutrophils [17]. The increased hydrophobicity of the ester also altered its antioxidant activity [17], [18]. Hence, we hypothesized that esterification could also alter its pro-oxidant potency. For this reason, we synthesized and studied esters of protocatechuic acid. The results confirmed our hypothesis, because greater hydrophobicity resulted in an increased and selective pro-oxidant activity. Besides, this work provides the first study on the specific and potent pro-oxidant activity of a series of protocatechuic acid alkyl esters for hydroquinone and related moieties and target biomolecules.

## Materials and Methods

### Chemicals

Protocatechuic acid, ethyl protocatechuate, gallic acid, butyl gallate, 3,5-dihydroxybenzoic acid, apocynin, trolox, α-tocopherol, glutathione (GSH), rifampicin, horseradish peroxidase (HRP), reduced nicotinamide adenine dinucleotide (NADH), diethylenetriaminepentaacetic acid (DTPA), 2,2′-Azino-bis(3-ethylbenzthiazoline-6-sulfonic acid) (ABTS), catalase, 5,5′-dithiobis(2-nitrobenzoic acid) (DTNB) and *N*,*N*′-dicyclohexylcarbodiimide were purchased from Sigma-Aldrich Chemical Co. (St. Louis, MO, USA). H_2_O_2_ was prepared by diluting a 30% stock solution and calculating its concentration from its absorbance at 240 nm (ε_240_ = 43.6 M^−1^ cm^−1^). All reagents used for buffers and mobile phases were of analytical grade. Stock solutions (50 mM) of the tested compounds were prepared in ethyl alcohol. Stock solution (10 mM) of α-tocopherol was prepared in ethyl alcohol. Ultrapure Milli-Q water from Millipore (Belford, MA, USA) was used for the preparation of buffers and solutions.

### Preparation of alkyl protocatechuates

The esters of protocatechuic acid were prepared as recently described [17]. A 3 mL solution of *N*,*N*′-dicyclohexylcarbodiimide (DCC, 1.0 mmol) in *p*-dioxane was added to a cooled (5°C) solution of 0.2 mmol protocatechuic acid and 20 mmol of butyl, heptyl, or decyl alcohols in 6 mL *p*-dioxane. The solution was stirred for 48 hours and the solvent was removed under reduced pressure. The residue was then partitioned 3 times with ethyl acetate and filtered. The filtrate was washed successively with saturated aqueous citric acid solution (3 times), saturated aqueous NaHCO_3_ (3 times), water (2 times), dried over anhydrous MgSO_4_, and evaporated under reduced pressure. The crude products were purified over a silica gel column (0.06–0.20 mm, ACROS Organics, USA) and eluted isocratically with CHCl_3_–CH_3_OH (98∶2). All compounds were identified using ^1^H and ^13^C NMR spectral data obtained from a Varian DRX-500 spectrometer (11.7 T). Chemical shifts (*δ*) were expressed in ppm. Coupling constants (*J*) were expressed in Hz, and splitting patterns are described as follows: s =  singlet; br s =  broad singlet; d =  doublet; t =  triplet; m =  multiplet; and dd =  doublet of doublets.

Butyl protocatechuate. It was obtained as a white solid in 93% yield. ^1^H NMR (500 MHz, DMSO-*d*_6_): *δ* 0.93 (t, *J* = 6.5 Hz, 3H), 1.30–1.82 (m, 4 H), 4.23 (t, *J* = 6.5 Hz, 2H), 6.79 (d, *J* = 8.5 Hz, 1H), 7.29 (dd, *J* = 2.0, 8.5 Hz, 1H), 7.34 (d, *J* = 2.0 Hz, 1H). ^13^C NMR (125 MHz, DMSO-*d*_6_) *δ* 13.5, *δ* 18.7, *δ* 30.3, *δ* 63.8, *δ* 165,6, *δ* 112.5, *δ* 123.3, *δ* 151.5, *δ* 147.4, *δ* 120.8, *δ* 115.2.

Heptyl protocatechuate. It was obtained as a white solid in 89% yield. ^1^H NMR (500 MHz, DMSO-*d*_6_): *δ* 0.84 (t, *J* = 6.5 Hz, 3H), 1.20–1.70 (m, 10 H), 4.15 (t, *J* = 6.5 Hz, 2H), 6.79 (d, *J* = 8.5 Hz, 1H), 7.29 (dd, *J* = 2.0, 8.5 Hz, 1H), 7.34 (d, *J* = 2.0 Hz, 1H). ^13^C NMR (125 MHz, DMSO-*d*_6_) *δ* 13.9, *δ* 22.0, *δ* 25.5, *δ* 28.2, *δ* 28.3, *δ* 31.2, *δ* 64.0, *δ* 165,7, *δ* 116.2, *δ* 121.7, *δ* 150.4, *δ* 145.1, *δ* 120.8, *δ* 115.3.

Decyl protocatechuate. It was obtained as a yellow solid in 73% yield. ^1^H NMR (500 MHz, DMSO-*d*_6_): *δ* 0.87 (t, *J* = 6.5 Hz, 3H), 1.25–1.76 (m, 16 H), 4.17 (t, *J* = 6.5 Hz, 2H), 6.79 (d, *J* = 8.5 Hz, 1H), 7.29 (dd, *J* = 2.0, 8.5 Hz, 1H), 7.34 (d, *J* = 2.0 Hz, 1H). ^13^C NMR (125 MHz, DMSO-*d*_6_) *δ* 13.7, *δ* 22.1, *δ* 25.3, *δ* 28.2, *δ* 28.3, *δ* 28.2, *δ* 28.3, *δ* 29.5, *δ* 31.3, *δ* 64.0, *δ* 165,7, *δ* 116.2, *δ* 121.7, *δ* 150.4, *δ* 145.1, *δ* 120.8, *δ* 115.3.

### Electrochemical measurements

Voltammetric studies were performed and oxidation potential, measured as anodic peak potential (Epa), was determined using an Autolab PGSTAT 30 potentiostat/galvanostat (Eco-Chemie, Utrecht, Netherlands). Voltammetric curves were recorded at room temperature using a 3-electrode setup cell. The working electrode was a glassy carbon disk electrode (GC electrode, 3 mm diameter). The counter electrode was a platinum plate and the reference an Ag/AgCl electrode saturated with 3 M KCl. The working electrode surface was carefully polished with 0.5 µm alumina slurries before each experiment and thoroughly rinsed with distilled water. A solution of sodium phosphate buffer 0.2 M (pH = 7) was used as a supporting electrolyte. The solutions were purged with nitrogen for 5 min before recording the voltammograms. The ethanolic solutions (5 mM) of the compounds were diluted in the electrochemical cell to final concentrations of 0.1 mM using the supporting electrolyte solution. The cyclic voltammograms were recorded at a potential scan rate of 5 mV s^−1^. Differential pulse voltammograms were obtained at modulation time 0.05 s, pulse amplitude 0.05 V, step potential 0.45 mV and scan rate 4.5 mV/s [19].

### Oxidation of trolox

A 100 µM trolox solution was incubated with 50 µM H_2_O_2_ and 0.01 µM HRP in 50 mM phosphate buffer, pH 7.0, with 50 µM DTPA at 25°C in the absence (control) or presence of the test compounds. The rate of trolox oxidation was measured at 272 nm. The blank for absorbance measurements consisted of phosphate buffer and the same concentration of the tested compounds. An HP8452 diode array spectrophotometer (Agilent, SC, CA, USA) was used to measure absorbance in these assays [20].

### Oxidation of α-tocopherol

A 100 µM α-tocopherol solution was incubated with 100 µM H_2_O_2_ and 0.01 µM HRP in 50 mM phosphate buffer, pH 7.0, with 50 µM DTPA at 25°C in the absence (control) or presence of the test compounds. The reactions were stopped after 10 min by the addition of 10 µg/mL catalase and an aliquot of 50 µL was injected into the HPLC. The analyses were carried out on a Luna C18 reversed-phase column (250×4.6 mm, 5 µm) using an HPLC–PDA detection system set at 295 nm (Jasco, Easton, MD, USA). The mobile phase consisted of 95% methanol and 5% water and the flow rate was 1.2 mL/min.

### Oxidation of rifampicin

A 100 µM rifampicin solution was incubated with 100 µM H_2_O_2_ and 0.01 µM HRP in 50 mM phosphate buffer, pH 5.5, at 25°C in the absence (control) or presence of the test compounds. The rate of rifampicin oxidation was measured at 472 nm. The blank for absorbance measurements consisted of phosphate buffer. An HP8452 diode array spectrophotometer (Agilent, SC, CA, USA) was used to measure absorbance in these assays [21].

### Oxidation of Glutathione

A 1 mM GSH solution was incubated with 100 µM H_2_O_2_ and 0.1 µM HRP in 50 mM phosphate buffer, pH 7.0 at 25°C in the absence (control) or presence of the test compounds. The reactions were stopped after 5 min by the addition of 10 µg/mL catalase. The concentration of GSH remaining was measured using the DTNB method, as previously described [22]. Briefly, the supernatant (0.45 mL) was combined with an equal volume of 300 mM Na_2_HPO_4_ and 0.1 mL of DTNB solution (0.2 mg/mL DTNB in 1% citrate). The absorbance at 412 nm was calculated relative to a blank containing 0.45 mL PBS, 0.45 mL 300 mM Na_2_HPO_4_, and 0.1 mL DTNB. A standard curve was generated to calculate the concentration of GSH. An HP8452 diode array spectrophotometer (Agilent, SC, CA, USA) was used to measure absorbance in these assays.

### Oxidation of NADH

A 100 µM NADH solution was incubated with 10 µM H_2_O_2_ and 0.01 µM HRP in 50 mM phosphate buffer, pH 7.0, with 50 µM DTPA at 25°C in the absence (control) or presence of the test compounds. The rate of NADH oxidation was measured at 340 nm. The blank for absorbance measurements consisted of phosphate buffer and the same concentration of the tested compounds. An HP8452 diode array spectrophotometer (Agilent, SC, CA, USA) was used to measure absorbance in these assays [20].

### Statistical analysis

The assays were conducted in duplicate and on three different days using different solutions. The results are expressed as mean ± SEM. Analysis of variance and significant differences among the means were tested by one-way ANOVA and Student-Newman-Keuls Multiple Comparison Test. Values of p<0.05 were regarded as significant.

## Results and Discussion

### Pro-oxidant activity (trolox as the target)

**Figure 1:**
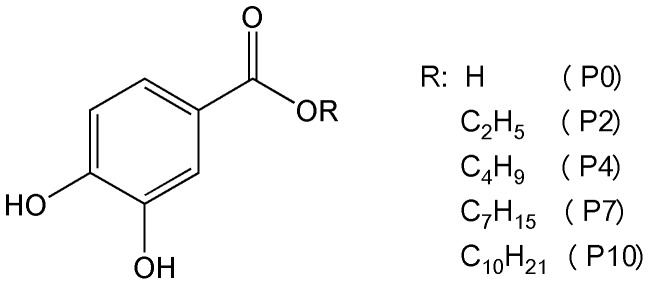
Molecular structures of protocatechuic acid and its alkyl esters.

**Figure 2:**
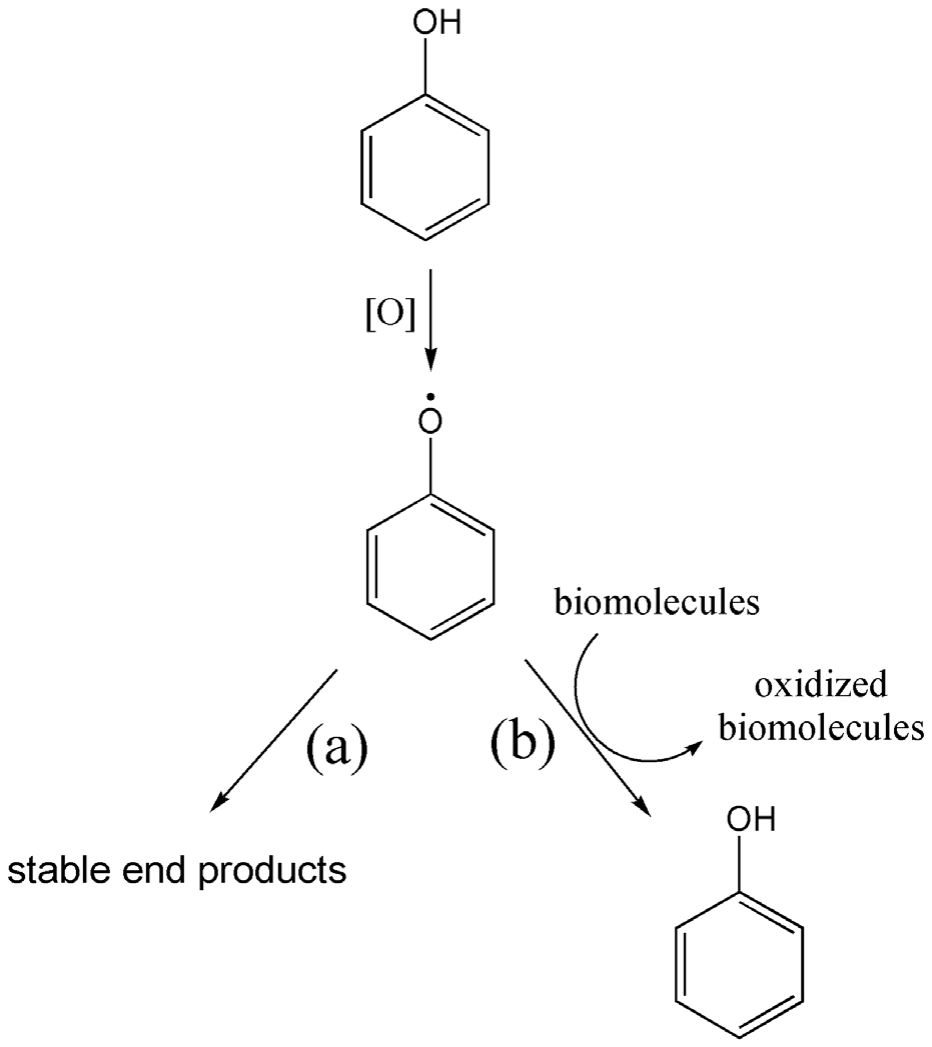
General pathways for phenol derivatives as (a) antioxidant or (b) pro-oxidant.

The molecular structures of protocatechuic acid (**P0**) and its esters bearing alkyl chains containing two (**P2**), four (**P4**), seven (**P7**) and ten (**P10**) carbons are shown in Figure 1. We found that, in addition to the improved antiradical capacity [17], [18], [23], the esterification of **P0** was also crucial for its action as a pro-oxidant compound. First of all, it must be emphasized that for phenolic and other redox active compounds, the distinction between anti- and pro-oxidant effects might be related to the stability and reactivity of the transient phenoxyl radicals generated during their oxidation. In this regard, an antioxidant effect is obtained when the transient free radical undergoes subsequent reactions leading to stable and non-harmful end products. On the other hand, a pro-oxidant effect will be observed when the transient free radical is able to promote downstream oxidations. To reinforce this important concept, which is the major point in this report, the chemical equations in Figure 2 illustrate the action of a generic phenolic compound acting either as an antioxidant (pathway **a**) or a pro-oxidant (pathway **b**). Consistent with pathway (**b**), the pro-oxidant activity of transient free radicals has been demonstrated in many publications; for instance: oxidation of NADH by raloxifene radicals [24], oxidation and depletion of GSH in HL-60 cells by etoposide radicals [25], and the induction of microsomal lipid peroxidation, ascorbate and GSH by phenoxyl radicals of vitamin E and its analogues [26]. On the other hand, most of the phenolic acids, which usually act as antioxidants, follow the pathway (**a**).

Following this concept, our experimental approach was to assess the pro-oxidant activity of transient phenoxyl radicals of protocatechuic acid and its esters. The phenoxyl radicals were produced by their reactions with H_2_O_2_ in a reaction catalysed by HRP. This is a well-established procedure for the generation and study of the pro-oxidant capacity of phenoxyl radicals [20]–[21], [24]–[26]. In this experimental model, it is important that the target biomolecules that are subjected to the pro-oxidative effect either do not react, or react with low efficiency, with the HRP/H_2_O_2_ enzymatic system. This is the case for trolox, a water-soluble derivative of vitamin E that was one of the targets in our studies.

**Figure 3:**
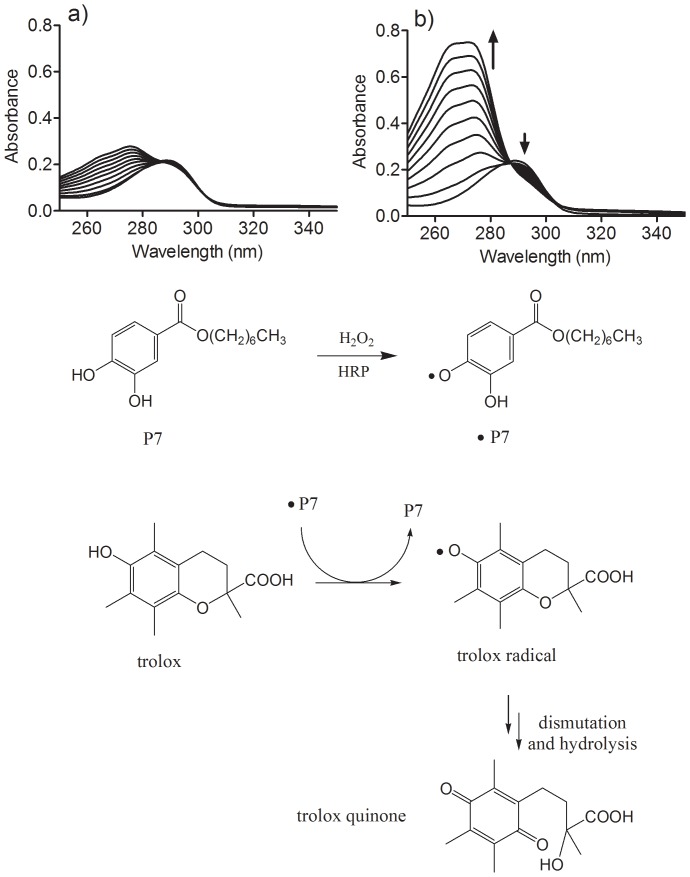
Effect and proposed pathway for the pro-oxidative action of P7 on trolox oxidation. The reaction mixture consisted of 100 µM trolox, 0.01 µM HRP and 50 µM H_2_O_2_ in the absence (a) or presence (b) of 2 µM P7. Scans were obtained at 5 s intervals.

The results depicted in Figure 3 show time-dependent changes in the absorbance of trolox when subjected to oxidation by HRP/H_2_O_2_ in the absence or presence of **P7**. It can be observed that the addition of a catalytic amount of **P7** provoked a significant increase in the rate of oxidation of trolox, which is indicative of its co-catalytic effect in this oxidation. It is noteworthy that the absorption band with a maximum at 272 nm and an isosbestic point at 286 nm is consistent with the formation of trolox quinone, which is the product of dismutation and hydrolysis of the transient trolox radical, as previously demonstrated [27], [28]. Figure 3 also shows our proposal for the effect of **P7** on trolox oxidation, consistent with a typical pro-oxidant effect.

**Figure 4:**
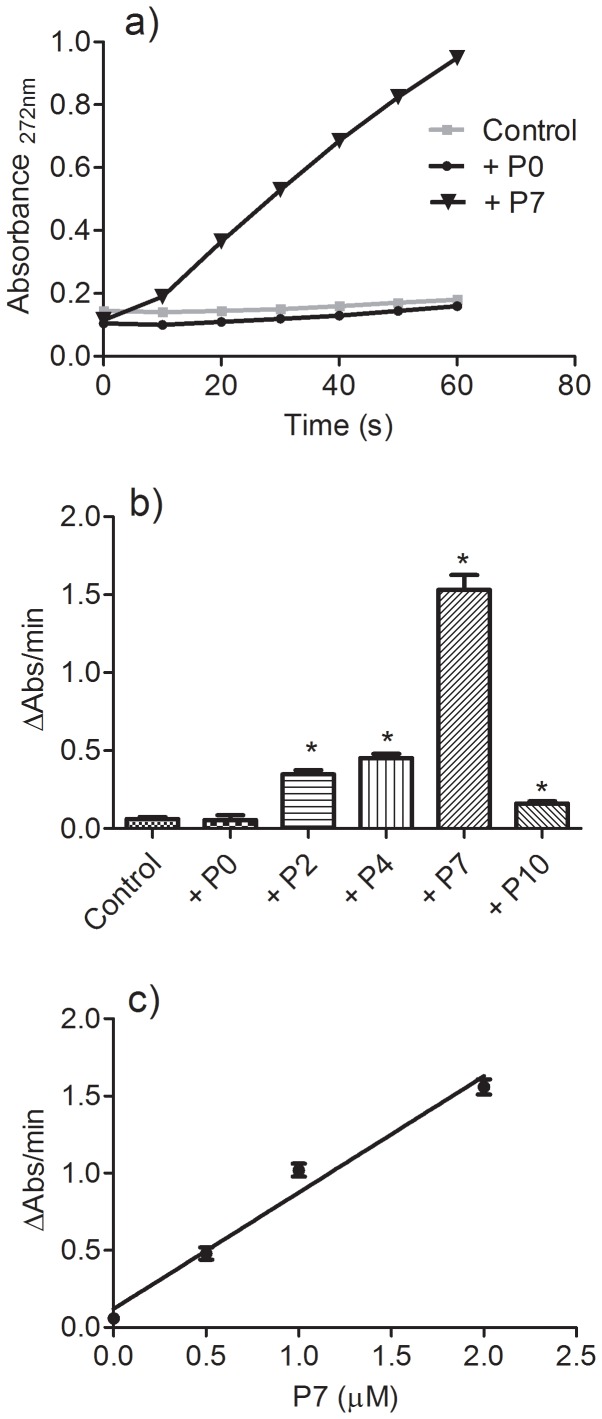
The length of the protocatechuate's carbon chain and the rate of oxidation of trolox. (a) The reaction mixture consisted of 100 µM trolox, 0.01 µM HRP and 50 µM H_2_O_2_ in the absence (control) or presence (2 µM) of the tested compounds. (b) Rate of oxidation. Data represent the mean and SEM of three experiments. *p<0.05 relative to the control. (c) Effect of the concentration of P7 on the rate of oxidation (r^2^  = 0.9766).

Unlike with **P7**, the results depicted in Figure 4[A] and 4[B] show that the addition of **P0** did not alter the oxidation of trolox. Moreover, except for **P10**, the pro-oxidant capacity was dependent on the length of the carbon chain, reaching its maximum with **P7**. The reduced efficacy of **P10** in this pro-oxidative model can be explained by its lower aqueous solubility, which decreases its effective concentration in the medium. The pro-oxidant effect of **P7** was also dependent on its concentration (Figure 4[C]). It is noteworthy that using 100 µM trolox and only 0.5 µM **P7**, the rate of oxidation of trolox was increased about eight-fold, which is a clear indication that **P7** was not consumed during the reaction course, hence confirming its co-catalytic effect in the oxidation of trolox.

**Figure 5:**
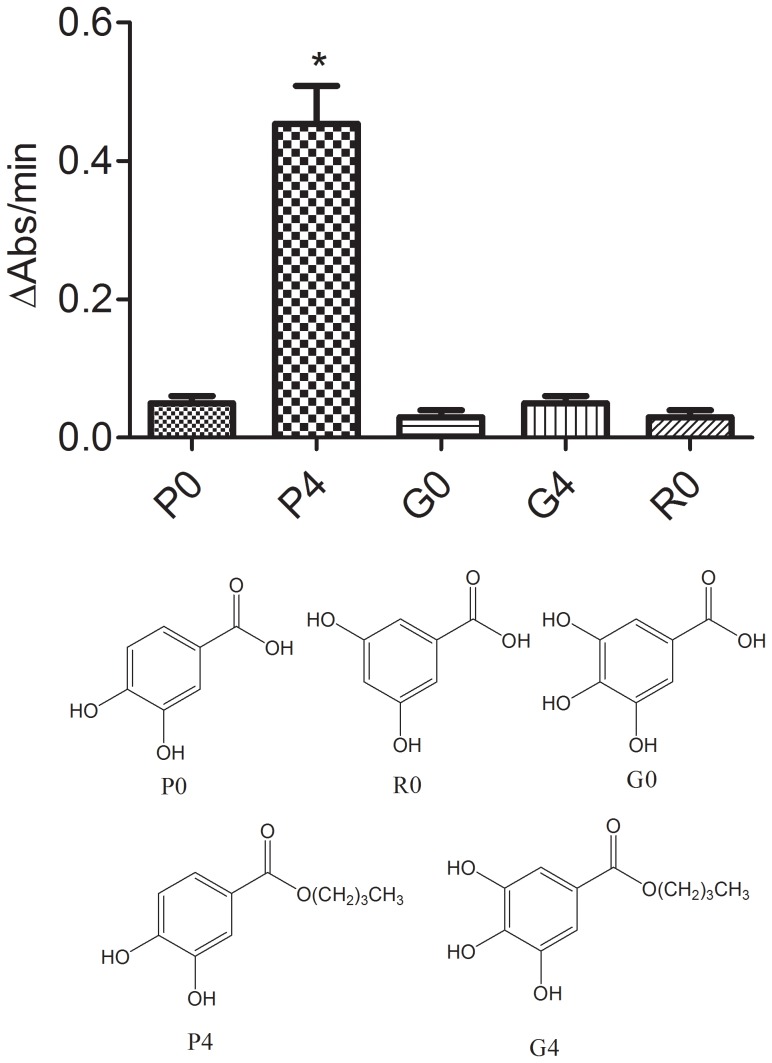
Effect of the number and position of hydroxyl groups and hydrophobicity on the rate of oxidation of trolox. The reaction mixture consisted of 100 µM trolox, 0.01 µM HRP and 50 µM H_2_O_2_ and 2 µM of the tested compounds. Data represent the mean and SD of three experiments. *p<0.05 relative to the P0.

The next step was to test the importance of the catechol moiety for the pro-oxidant effect. For that, we compared **P0** with gallic acid (**G0**) and the acid derivative of resorcinol (**RO**) (Figure 5). These compounds were chosen because there is a relationship between the number and position of hydroxyl groups in the benzene ring and the value of the oxidation potential. For instance, a recent study using a modified DNA-based glassy carbon electrode reported the following values: pyrogallol (0.39 V), catechol (0.42 V) and resorcinol (0.9 V) [29], which are related to **G0**, **P0** and **R0**, respectively. Hence, a comparison among **G0**, **P0** and **R0** could indicate the relationship between pro-oxidant activity and oxidation potential. From the results described in Figure 5, we concluded that the oxidation potential was not relevant to the pro-oxidant effect, since neither **G0** nor **R0** was able to act as a co-catalyst in the oxidation of trolox.

From another perspective and considering the importance of the esterification to the pro-oxidant activity of the tested compounds, we also measured and compared the pro-oxidant activities of butyl gallate (**G4**) and the equivalent **P4** (Figure 5). However, it can be seen that only **P4** was able to promote a co-catalytic effect. Hence, we concluded that the presence of the catechol moiety and the increased hydrophobicity are both necessary for the pro-oxidative activity demonstrated here.

### Pro-oxidative activity (GSH and NADH as targets)

**Figure 6:**
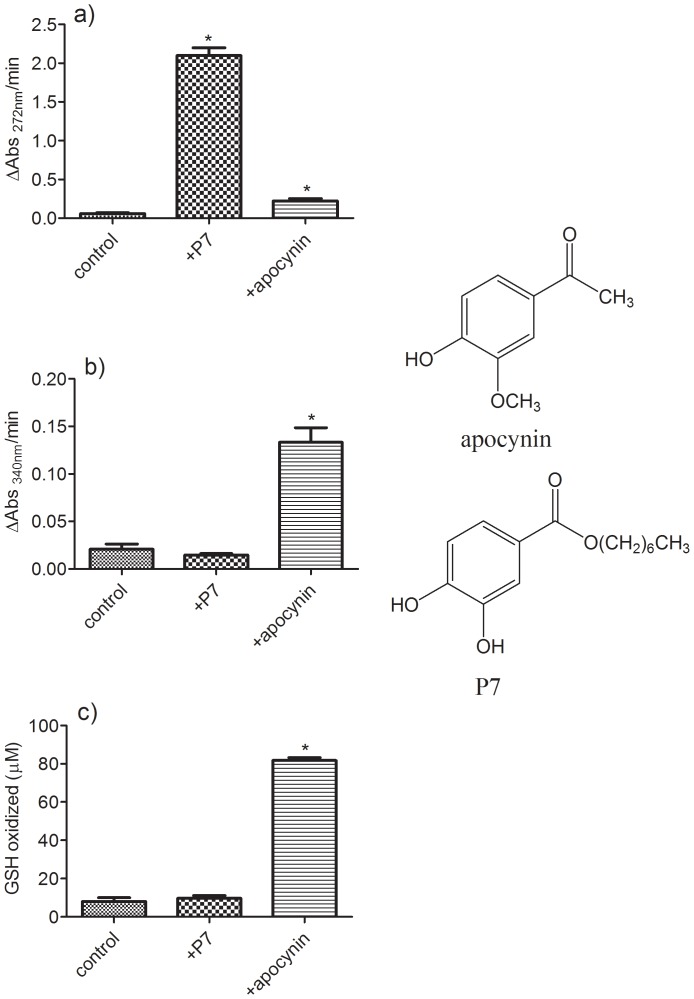
Apocynin versus P7 as pro-oxidants. (a) Oxidation of trolox, (b) oxidation of NADH and (c) oxidation of GSH. When present, apocynin and P7 were added at 5 µM. See material and methods for further experimental details. Data represent the mean and SD of three experiments. *p<0.05 relative to control.

So far, we have demonstrated that transient radicals generated during the oxidation of alkyl protocatechuates are able to oxidize trolox. However, besides trolox, several biomolecules have been identified as potential targets for pro-oxidative effects of transient radicals [21]–[32]. Hence, we studied the capacity of **P7** as a pro-oxidant of GSH and NADH and compared its efficacy with that of apocynin, a methoxy-catechol widely used as an inhibitor of the multienzymatic NADPH oxidase complex, and that has also been used as an anti-inflammatory [33], neuroprotective [34], and vasorelaxant [35], among many other biological applications. The apocynin radical is able to oxidize trolox, GSH, NADH and sulfhydryl residues in proteins [20]. The results depicted in Figure 6 show that apocynin was significantly more potent than **P7** as a pro-oxidant of NADH and GSH; however, **P7** was more effective regarding the oxidation of Trolox. These results suggested that the pro-oxidative activity of **P7** could be selective for the presence of hydroquinone or related functional group in the target molecules. These are relevant findings, since a specific pro-oxidant action could be useful as a modulator for quinone-base chemotherapeutics, where redox reactions are responsible by generation of cytotoxic oxidant species [36].

### Pro-oxidative activity (rifampicin and α-tocopherol as targets)

**Figure 7:**
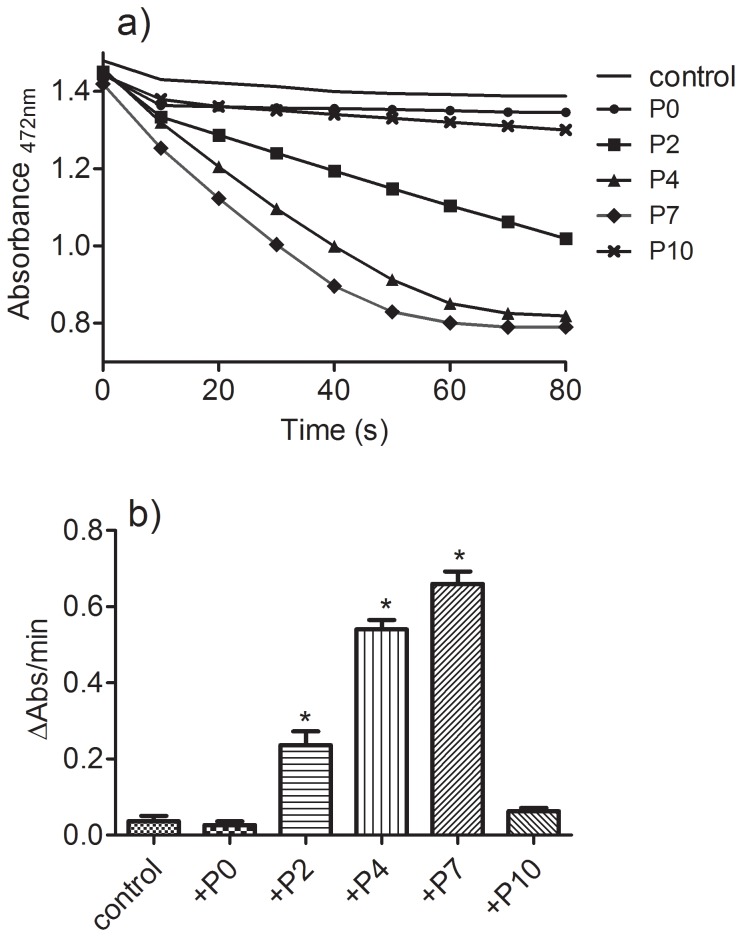
The length of a protocatechuate's carbon chain and the rate of oxidation of rifampicin. (a) The reaction mixture consisted of 100 µM rifampicin, 0.01 µM HRP, and 50 µM H_2_O_2_ in the absence (control) or presence (2 µM) of the tested compounds. (b) Reaction rate. Data represent the mean and SD of three experiments. *p<0.05 relative to the control.

Considering the above results, we studied the effect of the test compounds as pro-oxidants using rifampicin as target. The reason for the choice of this antibiotic was the presence of a hydroquinone moiety in its molecular structure, low reactivity with H_2_O_2_/HRP and the ease of monitoring its oxidation at 472 nm [21]. The results depicted in Figure 7 confirmed our expectation, since the addition of catalytic amount of the esters increased the rate of rifampicin oxidation significantly and, again, **P7** was the most effective compound.

**Figure 8:**
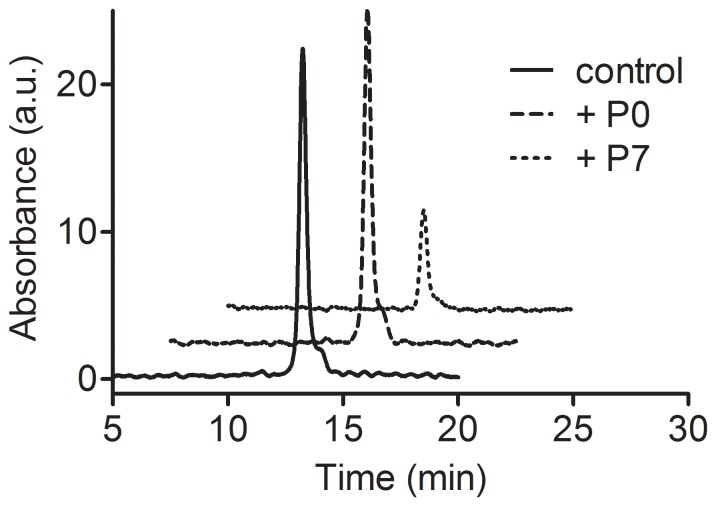
Oxidation of α-tocopherol and the effect of P0 and P7. The reaction mixture consisted of 100 µM α-tocopherol, 0.01 µM HRP, 50 µM H_2_O_2_ and 5 µM of the tested compounds. The reactions were stopped after 10 min and injected into the HPLC. See materials and methods for further experimental details. The control is a standard of α-tocopherol (100 µM).

For an additional confirmation of the pro-oxidant effect of the protocatechuates, α-tocopherol, the antioxidant present in cell membranes, was also used as a target. In this case the reaction was performed in the presence or absence of **P7** and after 10 minutes the reaction mixture was analysed by HPLC. Figure 8 shows that α-tocopherol was totally non-reactive with H_2_O_2_/HRP alone or after the addition of **P0**; however, the addition of **P7** caused its almost complete oxidation.

### Pro-oxidant activity and oxidation peak potential

It has been demonstrated that the capacity of phenolic compounds as pro-oxidants is partly correlated with the one-electron redox potential of their phenoxyl radicals [37]. For instance, naringenin (Epa  = 0.600 V) was about five-fold more efficient than rutin (Epa  = 0.180 V) as a co-catalyst in the oxidation of NADH by HRP/H_2_O_2_ [37]. Similarly, apocynin (Epa  = 0.760 V [38]) was four-fold more efficient than the structurally related compound vanillic acid (Epa  = 0.494 V, [39]) as a co-catalyst in the oxidation of GSH mediated by HRP/H_2_O_2_ [20]. Hence, it was not surprising to find that apocynin was more effective than **P7** (Epa  = 0.266 V) regarding co-oxidation of NADH and GSH (Figure 6). However, the relationship between the oxidation peak potential and pro-oxidant activity did not explain the results obtained for the oxidation of trolox, rifampicin and α-tocopherol, since **P7** was more efficient than apocynin with these compounds. These findings and the large difference between **P7** and **P0** in oxidizing the hydroquinone related compounds motivated us to conduct a more detailed study of the effect of esterification on the oxidation peak potential of protocatechuic acid.

**Figure 9:**
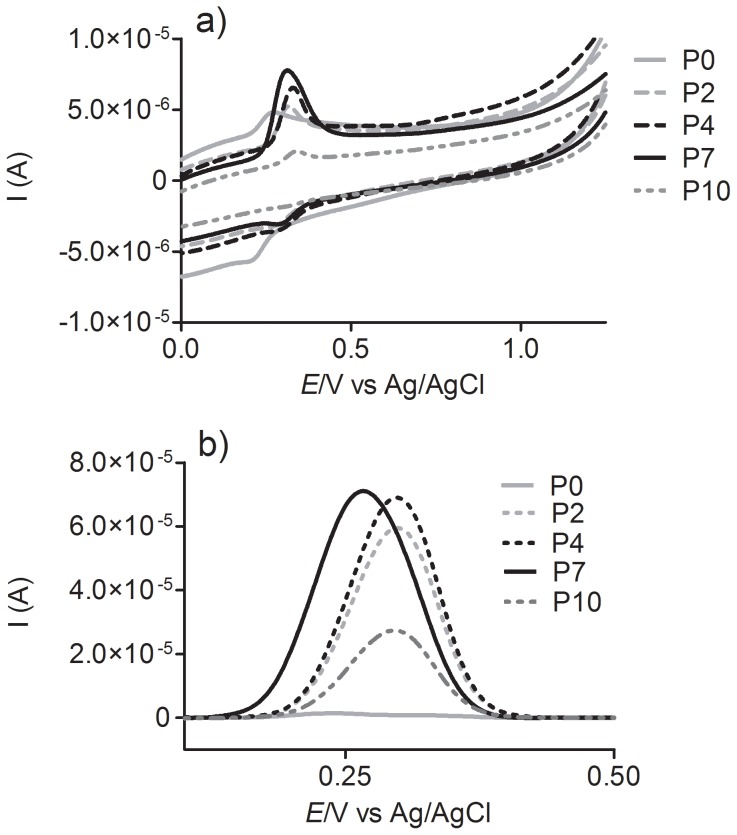
Cyclic (a) and differential pulse voltammograms (b) of 0.1 mM solutions of protocatechuic acid and its esters at pH 7.0. The cyclic voltammograms were recorded at a potential scan rate of 5 mV s^−1^. Differential pulse voltammograms were obtained at modulation time 0.05 s, pulse amplitude 0.05 V, step potential 0.45 mV and scan rate 4.5 mV/s.

The test compounds were studied looking for a relationship between their pro-oxidative capacities and their oxidation peak potential. Figure 9 shows the results of electrochemical experiments obtained using a glassy carbon working electrode at pH 7.0. The cyclic and differential pulse voltammetry experiments showed a single and well-defined oxidation peak for the protocatechuic alkyl esters, which ranged from 0.266 to 0.298 V. Similar values were reported by Reis et al. [18], who studied the relationship between antioxidant activity and the electrochemical properties of protocatechuic acid and its methyl, ethyl and propyl esters. Actually, the absence of a significant difference in the oxidation peak potentials for these compounds was not unexpected, since the esterification of a carboxylic group does not provoke a significant alteration in its redox potential. Comparisons can also be made between gallic acid (half-wave potential, E_1/2_, 0.52 V) and its esters, propyl gallate (E_1/2_ 0.51 V) and butyl gallate (E_1/2_ 0.51 V) [40]; caffeic acid (Epa 0.183 V) and ethyl caffeate (Epa 0.175 V); and ferulic acid (Epa 0.335 V) and ethyl ferulate (Epa 0.368 V) [41].

By comparing Epa values, it can be concluded that oxidation peak potential cannot explain the large and significant differences seen in the pro-oxidant effect. However, the same cannot be said for the intensity of the peak current as obtained in the differential pulse voltammetry experiments (Figure 9[B]). Here it can be observed that the peak intensity correlated with the pro-oxidant activity, since **P7** produced the higher current, whereas **P0** had the lowest.

As is well known, in differential pulse voltammetry experiments the peak current is related to the concentration, mass transport, chemical properties of the studied compound and physicochemical properties of the electrode. Hence, considering that the concentration of the tested compounds and the electrode properties were the same, only mass transport or the physicochemical properties of the analyte could be responsible for the differences observed in peak current. However, mass transport, which is proportional to the diffusion coefficient and, most straightforwardly, to the size of the molecules under investigation, seems to be very similar for each molecule, since **P0**, **P2**, **P4** and **P7**, although very different in molecular volumes, present similar peak current values. Hence, we suggest that the higher peak current intensity for **P2**, **P4** and **P7** must be related to the stability (lifetime) of the particular transient phenoxyl radical formed during the oxidation process, which could affect its efficacy as an oxidant species. An additional point must be considered regarding the relationship between the peak current intensity and pro-oxidative activity. How can be observed by comparing the Figure 4 and 9, the difference in the peak current intensity for **P2**, **P4** and **P7** was not proportional to their efficacy as pro-oxidant. This is an indication that additional factors must be involved in the efficacy of the esters compared to protocatechuic acid. However, there is no doubt that esterification was the most important factor.

**Figure 10:**
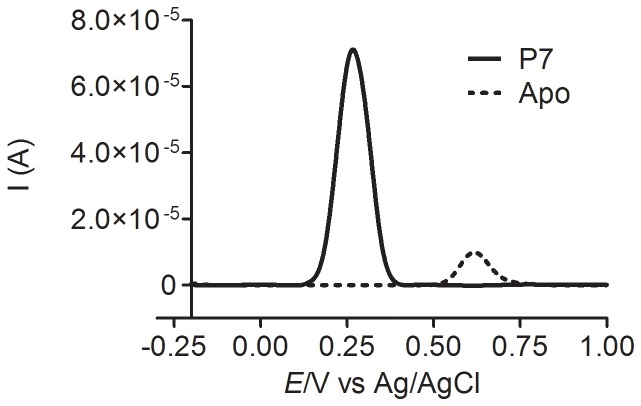
Differential pulse voltammograms of 0.1 mM solutions of heptyl protocatechuate and apocynin at pH 7.0. Differential pulse voltammograms were obtained at modulation time 0.05 s, pulse amplitude 0.05 V, step potential 0.45 mV and scan rate 4.5 mV/s.

It is noteworthy that in a classical peroxidase-catalysed reaction the substrates are oxidized by the active redox forms of the peroxidase, named compound I and II; then, the formed transient radicals diffuse from the enzyme active site and undergo subsequent reactions leading to dimers, trimers and higher oligomers [42]. Hence, taking into account these facts: i) the higher stability of the transient radicals of the esters of protocatechuic acid suggested by the differential pulse voltammetry experiments; and ii) the classical pathway for its formation through HRP-catalysed oxidation, the following proposal could explain our findings. Namely, after its formation in the active site of the enzyme and due to its higher lifetime, the radicals of **P2**, **P4** and **P7** could diffuse and interact with the target molecule, promoting its oxidation. On the other hand, the unstable **P0** radical would follow the usual pathway for free radicals, i.e. dimerization, oligomerization, etc. To reinforce this proposal, we also measured the differential pulse voltammetry of apocynin under the same experimental conditions. Figure 10 shows the results for **P7** and apocynin. Here it can be observed that, despite its higher oxidation peak potential, the intensity of the peak current for apocynin was significantly lower compared to **P7** and, as demonstrated above, less pro-oxidant.

## Conclusions

The involvement of transient pro-oxidant free radicals as deleterious species has long been recognized. For instance, the oxidation of LDL mediated by MPO-generated paracetamol radicals [43], the depletion of GSH by AAPH-induced melatonin oxidation [44], the oxidation of NADH by raloxifene radical [24], the oxidation and depletion of GSH in HL-60 cells by etoposide radicals [25], the induction of microsomal lipid peroxidation, ascorbate and GSH by phenoxyl radicals of vitamin E and analogues [26], etc. In this context, the magnitude of the oxidation peak potential is usually accepted as the major factor determining whether the transient free radicals will act as a pro-oxidant or antioxidant substance [45]. Herein, we have demonstrated for the first time that besides the magnitude of the oxidation potential, hydrophobicity can also be an important factor. Our findings that esters of protocatechuic acid are specific and potent co-catalysts in the oxidation of hydroquinone related compounds may be very relevant, since this moiety is wide present in biomolecules (ubiquinones) and pharmaceuticals (quinone-based chemotherapeutics). Hence, its interaction with hydroquinones could exacerbate redox cycling reactions that are involved in their biological and pharmacological mechanisms of action.
